# Impact of Renin-Angiotensin-Aldosterone System Modulation on Cognitive and Neuropsychiatric Outcomes: A Systematic Review of Clinical and Mechanistic Evidence

**DOI:** 10.7759/cureus.87557

**Published:** 2025-07-08

**Authors:** Muhammad Kamal Subhani, Astrit Sabani, Osman Omer, Fatima Kheirelsid, Abeer K Srour

**Affiliations:** 1 Internal Medicine, Mayo Hospital, Lahore, PAK; 2 Medicine and Surgery, St. George's University School of Medicine, St. George's, GRD; 3 Integrative Medicine, Prince Mohammed Bin Abdulaziz Hospital, Riyadh, SAU; 4 Internal Medicine, University of Khartoum, Khartoum, SDN; 5 General Practice, Palestine Medical Complex, Ramallah, PSE

**Keywords:** ace inhibitors, alzheimer's disease, angiotensin receptor blockers, cognitive decline, dementia, hypertension, neuropsychiatric outcomes, renin-angiotensin-aldosterone system, systematic review

## Abstract

This systematic review explores the influence of the renin-angiotensin-aldosterone system (RAAS) on cognitive and neuropsychiatric outcomes, synthesizing evidence from clinical and preclinical studies investigating RAAS-modulating therapies such as angiotensin-converting enzyme inhibitors (ACEIs) and angiotensin receptor blockers (ARBs). A comprehensive literature search across four major databases yielded seven studies that met the inclusion criteria, including randomized controlled trials, observational studies, and one animal model experiment. The findings suggest that RAAS modulation, particularly through ARBs, may offer cognitive benefits in certain populations, especially those with hypertension, type 2 diabetes, or vascular risk factors. Some studies demonstrated improvements in executive function, memory, and global cognition, while others found no statistically significant effect on neurodegeneration or cognitive decline. Mechanistic insights indicate that RAAS-targeting interventions may reduce neuroinflammation, oxidative stress, and microvascular dysfunction, which are pathways implicated in cognitive impairment. Although promising, the evidence is varied in methodological quality and consistency, highlighting the need for future trials with cognitive endpoints as primary outcomes.

## Introduction and background

Dementia is a progressive neurodegenerative condition marked by the deterioration of cognitive functions, including memory, reasoning, and behavior. As populations age, the prevalence of dementia continues to rise globally, posing immense personal, societal, and economic burdens [1]. While Alzheimer’s disease is the most common form, the pathogenesis of dementia encompasses a complex interplay of neurodegenerative, vascular, metabolic, and inflammatory processes [2]. Increasingly, research has turned toward systemic contributors beyond the brain itself, among which the renin-angiotensin-aldosterone system (RAAS) has emerged as a key player with both pathophysiological and therapeutic relevance [3].

Traditionally recognized for its role in regulating blood pressure and fluid balance, RAAS also exerts significant influence within the central nervous system. It affects cerebral blood flow, neuroinflammation, oxidative stress, and the integrity of the blood-brain barrier, mechanisms that are all intricately linked to cognitive decline and neuropsychiatric disturbances [4]. Elevated central RAAS activity, particularly through the actions of angiotensin II on AT1 receptors, has been associated with neuronal damage and impaired cognitive processing [5]. Conversely, RAAS inhibition, achieved pharmacologically through angiotensin-converting enzyme inhibitors (ACEIs) and angiotensin receptor blockers (ARBs), has shown promise in ameliorating these effects. These observations suggest that RAAS modulation may serve as a dual-purpose approach, simultaneously targeting cardiovascular comorbidities and cognitive dysfunction [6].

Recent years have seen a surge of studies exploring this relationship. Clinical trials such as the RADAR [7] and ONTARGET studies [8], along with ancillary analyses from broader trials like Look AHEAD [9], have examined the cognitive outcomes associated with RAAS-targeting therapies. Meanwhile, preclinical investigations have illuminated the neuroprotective potential of RAAS blockade in models of vascular cognitive impairment, stroke, and neuroinflammation. Some studies also suggest that genetic variants within RAAS components may predispose individuals to dementia, offering further insights into the underlying biological mechanisms. However, despite these advances, the evidence remains fragmented. Variations in study populations, endpoints, treatment durations, and assessment methods have contributed to inconsistent conclusions regarding the efficacy and mechanisms of RAAS-targeting interventions in cognitive health.

In this context, the present systematic review seeks to synthesize the current body of evidence addressing the influence of the renin-angiotensin-aldosterone system on dementia and neuropsychiatric outcomes. Specifically, we aim to evaluate both the pathophysiologic mechanisms by which RAAS contributes to neurocognitive decline and the therapeutic potential of its pharmacologic modulation. The objective of this review is framed by the following PICO (Population, Intervention, Comparison, Outcome) question [10]: In adults or animal models at risk of or diagnosed with dementia or neuropsychiatric impairment (Population), does the use of RAAS-modulating agents such as ACE inhibitors or ARBs, or the measurement of RAAS activity (Intervention), compared to placebo, non-RAAS antihypertensive agents, or no treatment (Comparison), lead to improved cognitive function, delayed dementia progression, or reduced neuropsychiatric symptoms (Outcome)?

## Review

Materials and methods

Search Strategy

This systematic review was conducted in accordance with the PRISMA (Preferred Reporting Items for Systematic Reviews and Meta-Analyses) 2020 guidelines [11] to ensure transparency and methodological rigor. A comprehensive literature search was performed across multiple databases, including PubMed, Scopus, Web of Science, and the Cochrane Library. The search strategy incorporated controlled vocabulary (e.g., MeSH terms) and keywords such as “renin-angiotensin-aldosterone system,” “cognition,” “dementia,” “angiotensin receptor blockers,” “ACE inhibitors,” and “neuropsychiatric outcomes.” Boolean operators were employed to refine results, and filters were applied to restrict studies to those published in English and involving human or relevant animal models. Reference lists of key articles were also manually screened to identify any additional eligible studies.

Eligibility Criteria

Studies were included if they (1) investigated the association between RAAS-modulating therapies (e.g., ACE inhibitors, ARBs, or angiotensin receptor agonists) and cognitive or neuropsychiatric outcomes, (2) involved either human participants at risk of or diagnosed with cognitive impairment or animal models of neurodegeneration, and (3) were randomized controlled trials, observational studies, or preclinical experiments. Articles were excluded if they were protocols without results, narrative reviews, editorials, or studies that did not report cognitive or neuropsychiatric endpoints. The final selection was based on relevance to the research question and data availability for extraction and analysis.

Particular attention was paid to the potential for confounding in observational studies. During data extraction, we noted whether included studies adjusted for key confounders such as age, education, baseline cognitive status, comorbidities (e.g., diabetes, hypertension), and concurrent medications. The Newcastle-Ottawa Scale was used to evaluate selection bias, comparability of cohorts, and outcome ascertainment. Studies with limited adjustment or no control groups were interpreted with caution in the synthesis.

Data Extraction

For each eligible study, data were independently extracted by two reviewers using a standardized data collection form. Extracted variables included study design, sample size, population characteristics, type and dosage of RAAS intervention, duration of follow-up, comparator details, primary cognitive or neuropsychiatric outcomes assessed, and key statistical findings. In cases of disagreement, consensus was achieved through discussion or consultation with a third reviewer. Where necessary, authors of original studies were contacted for additional data clarification.

Data Analysis and Synthesis

Given the heterogeneity in study designs, populations, interventions, and cognitive assessment tools, a meta-analysis was not feasible. Instead, a qualitative narrative synthesis was employed to analyze and integrate findings across studies. Results were grouped according to study type (clinical trials, observational studies, preclinical trials) and nature of intervention (ARB, ACEI, or combination). Trends in efficacy, statistical significance of outcomes, and mechanistic insights were summarized to elucidate the role of RAAS modulation in cognitive and neuropsychiatric health. Studies were also compared based on methodological strengths, effect sizes where available, and relevance to clinical practice.

Results

Study Selection Process

**Figure 1:**
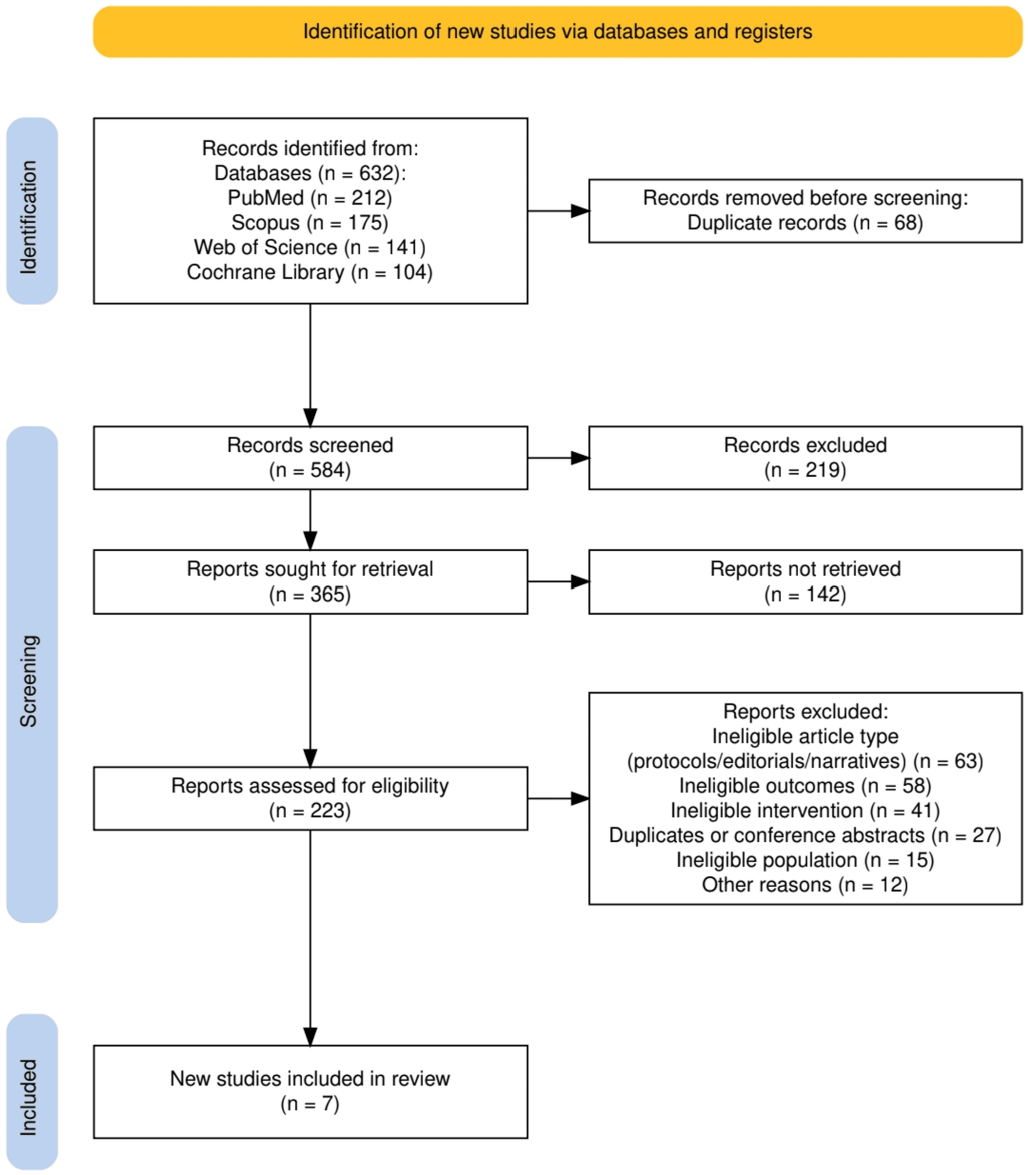
The PRISMA flowchart represents the study selection process. PRISMA: Preferred Reporting Items for Systematic reviews and Meta-Analyses

Based on the updated PRISMA diagram provided in Figure 1, the study selection process was conducted in alignment with the PRISMA 2020 guidelines to ensure transparency and methodological integrity. A total of 632 records were retrieved from four electronic databases: PubMed (n = 212), Scopus (n = 175), Web of Science (n = 141), and the Cochrane Library (n = 104). After the removal of 68 duplicate entries, 584 records remained for title and abstract screening. Of these, 219 records were excluded due to irrelevance to the topic. The full texts of 365 reports were sought for retrieval, out of which 142 could not be accessed. A total of 223 full-text articles were assessed for eligibility. Among them, 204 were excluded for the following reasons: protocols, narrative reviews, or editorials without results (n = 63); lack of relevant cognitive or neuropsychiatric outcomes (n = 58); interventions not involving RAAS-modulating agents (n = 41); non-original studies such as duplicates or conference abstracts (n = 27); and populations not meeting inclusion criteria (n = 15). Ultimately, 7 studies were deemed eligible and included in the final qualitative synthesis.

Characteristics of the Selected Studies

Table 1 summarizes the key characteristics of the seven studies selected for inclusion in this systematic review, encompassing a range of study designs, populations, interventions, and outcome measures relevant to the influence of the RAAS on cognitive function and neuropsychiatric outcomes. The majority of included studies were randomized controlled trials (RCTs), including both clinical and preclinical designs, while two were observational analyses. Study populations varied widely, from individuals with mild-to-moderate Alzheimer’s disease and type 2 diabetes to hypertensive patients and healthy older adults. Interventions predominantly involved ARBs such as losartan, candesartan, and eprosartan, with one study examining a selective AT2 receptor agonist and another evaluating ACE inhibitors in relation to genetic polymorphism. Treatment durations ranged from one week to over five years, with cognitive endpoints assessed via tools such as the Mini-Mental State Examination (MMSE), memory scales, executive function tests, and neuroimaging. The findings were heterogeneous: some studies reported significant cognitive benefits associated with RAAS modulation, particularly in executive function, memory performance, or cognitive preservation linked to blood pressure control, while others showed no statistically significant cognitive improvement. Mechanistically, the interventions targeted either AT1 receptor blockade, AT2 receptor stimulation, or ACE activity modulation, collectively offering insight into multiple RAAS-related pathways potentially influencing neurocognitive health.

**Table 1:** The summary of the studies included in the systematic review. RCT: randomized controlled trial; RAAS: renin-angiotensin-aldosterone system; RAAS-AHM: renin-angiotensin-aldosterone system–acting antihypertensive medication; ACEI: angiotensin-converting enzyme inhibitor; ARB: angiotensin II receptor blocker; AT1: angiotensin II type 1 receptor; AT2R: angiotensin II type 2 receptor; T2DM: type 2 diabetes mellitus; AD: Alzheimer’s disease; MRI: magnetic resonance imaging; CVD: cardiovascular disease; MMSE: Mini-Mental State Examination; C21: compound 21 (selective AT2R agonist); WMS-R: Wechsler Memory Scale-Revised; ACE I/D polymorphism: insertion/deletion polymorphism in the ACE gene; DD genotyp: homozygous deletion genotype of the ACE gene; T1DM: type 1 diabetes mellitus; EEG: electroencephalogram; BP: blood pressure; ↑: increase; ↓: decrease

Study (Author, Year)	Study Design	Population (N, Age, Condition)	Intervention (Type, Dose, Duration)	Comparator	Outcomes Measured	Key Findings	RAAS Mechanism Targeted
Kehoe et al., 2021 (RADAR) [7]	RCT, Phase 2	N = 211; ≥55 yrs; mild-to-moderate AD	Losartan 100 mg/day for 12 months	Placebo	Brain volume (MRI), cognitive decline	No significant difference in brain atrophy; well tolerated	ARB (AT1 receptor blockade)
Wharton et al., 2022 (Look AHEAD) [9]	Secondary analysis of RCT	N = 712; T2DM, overweight/obese adults	RAAS-AHM (ACEI/ARB); long-term	Other-AHM	Executive function, memory, composite cognition	Better executive function and memory in RAAS-AHM users	ACEI and/or ARB
Anderson et al., 2011 (ONTARGET/TRANSCEND) [8]	RCT, pooled analysis	N = 31,546; ≥55 yrs with CVD or diabetes	Ramipril, Telmisartan, Combo; ~56 months	Placebo, other RAS arms	MMSE decline, cognitive impairment	No significant differences; trend favoring telmisartan vs. ramipril	ACEI and ARB
Ahmed et al., 2018 [12]	Preclinical RCT (rats)	Hypertensive rats post-stroke	Candesartan or C21 for 30 days	Saline	Cognition, amyloid-β, microgliosis	Both treatments preserved cognition and reduced inflammation	ARB (AT1) and AT2R agonist
Schuch et al., 2014 [13]	Observational genetic study	N = 205; Healthy adults >50 yrs	ACEI use; ACE I/D polymorphism	Non-users; genotype (DD)	WMS-R memory scores	ACEI + I allele = better learning/verbal memory	ACE gene activity modulation
Færch et al., 2015 [14]	RCT, cross-over	N = 9; T1DM, high RAS activity	Candesartan 32 mg/day for 1 week	Placebo	Cognitive tests, EEG, hormonal responses	No cognitive effect during hypoglycemia; altered glucose handling	ARB (Candesartan)
Hanon et al., 2008 [15]	Open-label clinical trial	N = 25,745; Hypertensive ≥50 yrs	Eprosartan 600 mg/day for 6 months	None	BP change, MMSE	BP ↓ and MMSE ↑; greater BP reduction = better cognition	ARB (Eprosartan)

Quality Assessment

The quality assessment of the included studies, detailed in Table 2, reflects a spectrum of methodological rigor and risk of bias across different study designs. Among the randomized controlled trials (RCTs), two studies were evaluated using the Cochrane Risk of Bias 2.0 tool [16] and were judged to have a low risk of bias, owing to robust randomization procedures, blinding, and intention-to-treat (ITT) analyses. These included a well-powered multicenter trial and a phase 2 intervention study, both of which adhered closely to methodological best practices, although in one case, cognitive outcomes were secondary endpoints. One crossover RCT raised some concerns due to its small sample size (n = 9), which may limit generalizability despite the appropriateness of the design. The preclinical study assessed with SYRCLE’s tool [17] showed unclear risk, primarily because blinding methods were not well described, even though the experimental outcomes were clearly beneficial. Observational studies were appraised using the Newcastle-Ottawa Scale [18], with scores indicating moderate quality. While these studies offered large sample sizes and relevant outcome measures, they were limited by potential confounding factors, lack of control groups, or reliance on self-reported exposure data. Overall, the included studies provide a reasonably solid evidence base, though variability in study design and quality necessitates cautious interpretation of the collective findings.

**Table 2:** The risk of bias assessment of each of the included study.

Study (Author, Year)	Study Design	Tool Used	Risk of Bias / Quality Judgment	Comments
Kehoe et al., 2021 (RADAR) [7]	RCT	Cochrane RoB 2	Low Risk	Proper randomization, blinding, ITT analysis; primary outcome met; well-conducted
Wharton et al., 2022 (Look AHEAD) [9]	Secondary analysis of RCT	Newcastle-Ottawa Scale	Moderate Quality (7/9)	Large sample, robust stats; risk of residual confounding due to observational design
Anderson et al., 2011 (ONTARGET/TRANSCEND) [8]	RCT	Cochrane RoB 2	Low Risk	Large, multicenter RCT; cognitive outcome was secondary; minimal missing data
Ahmed et al., 2018 [12]	Preclinical RCT	SYRCLE RoB	Unclear Risk	Randomization reported; blinding less clear; strong outcomes but generalizability limited
Schuch et al., 2014 [13]	Observational genetic study	Newcastle-Ottawa Scale	Moderate Quality (6/9)	Adequate outcome measures; small sample; self-reported drug use may bias exposure data
Færch et al., 2015 [14]	Cross-over RCT	Cochrane RoB 2	Some Concerns	Small sample (N=9); crossover design appropriate; limited generalizability
Hanon et al., 2008 [15]	Open-label observational trial	Newcastle-Ottawa Scale	Moderate Quality (6/9)	Very large sample; no control group; outcome based on MMSE only

Discussion

Our review of seven studies assessing the impact of RAAS modulation on dementia and cognitive outcomes reveals a nuanced yet promising therapeutic potential. Among the included trials, ARBs such as losartan, candesartan, and eprosartan demonstrated variable but generally favorable effects on cognitive preservation. For instance, Wharton et al. [9] found significantly better executive function (p < 0.04), processing speed (p < 0.004), and verbal memory (p < 0.005) among RAAS-AHM users compared to those on other antihypertensives in adults with type 2 diabetes. Hanon et al. [15] reported an average increase in MMSE scores [19] from 27.1 to 27.9 (p < 0.0001) following six months of eprosartan therapy in over 25,000 hypertensive patients. Conversely, the RADAR trial by Kehoe et al. [7] found no statistically significant difference in brain atrophy between losartan and placebo groups after 12 months (mean difference -2.29 mL, 95% CI -6.46 to 0.89; p = 0.14), though treatment was well tolerated. Meanwhile, Anderson et al. [8] showed no clear cognitive advantage for telmisartan or ramipril over placebo, though a trend favoring telmisartan was noted (OR 0.90; p = 0.06). These mixed findings underscore both the therapeutic promise and the current limitations of RAAS-targeted strategies in dementia care.

From a pathophysiological standpoint, the RAAS plays a critical role in brain homeostasis through the regulation of cerebral perfusion, oxidative stress, and neuroinflammatory pathways. Angiotensin II, particularly via the AT1 receptor, contributes to endothelial dysfunction, increased blood-brain barrier permeability, and proinflammatory cascades, all of which are implicated in neurodegenerative processes [20]. Ahmed et al. [12] demonstrated that both candesartan and the selective AT2 receptor agonist compound-21 preserved cognitive function and reduced microgliosis in a hypertensive rat stroke model, independent of blood pressure effects or amyloid accumulation. This supports the hypothesis that RAAS blockade, especially when targeting AT1 inhibition and AT2 activation, offers neurovascular protection. Furthermore, Schuch et al. [13] highlighted a gene-drug interaction where individuals with the I allele of the ACE I/D polymorphism exhibited enhanced verbal memory only when using ACE inhibitors, suggesting that genetic predisposition may modulate the cognitive effects of RAAS intervention. These findings reinforce the concept that RAAS activity is not only systemically but also centrally relevant in the context of cognitive aging.

The clinical implications of RAAS modulation extend to a broad range of populations, particularly those with metabolic or vascular comorbidities [21]. In the Wharton et al. [9] study, individuals with type 2 diabetes, who are inherently at higher risk for both vascular dementia and Alzheimer’s disease, benefited cognitively from RAAS-targeting antihypertensives compared to other classes. Similarly, the large-scale observational trial by Hanon et al. (2008) found that greater reductions in systolic blood pressure (<140 mmHg) were associated with more substantial cognitive improvements, with mean MMSE gains of 0.88 points (p < 0.001). This supports the dual role of blood pressure management and RAAS-specific effects in promoting cognitive health. Notably, the ONTARGET study [8] found no significant differences between ramipril and telmisartan in terms of cognitive decline, but did observe a non-significant trend favoring telmisartan (OR 0.90), hinting at potential advantages of ARBs over ACEIs in central nervous system protection. Collectively, these studies suggest that patient-specific factors, such as comorbidity profile and choice of RAAS agent, may influence cognitive outcomes.

While this systematic review provides valuable insights into the cognitive and neuropsychiatric effects of RAAS modulation, several limitations must be acknowledged. The included studies demonstrated considerable heterogeneity in terms of populations, interventions, cognitive assessment tools, and outcome measures, which limited the ability to perform a meta-analysis and may affect the generalizability of the findings. In many cases, cognitive outcomes were secondary endpoints, potentially underpowered to detect meaningful changes and increasing the risk of outcome reporting bias. For example, although the RADAR [7] and ONTARGET [8] trials were large and well-conducted, cognitive outcomes were not primary endpoints, reducing their sensitivity to detect cognitive changes. The Færch et al. study [14], a crossover RCT with only nine participants, also limits external validity despite its appropriate design. Additionally, the inclusion of both clinical and preclinical studies, such as the mechanistically informative animal model by Ahmed et al. [12], introduces complexity in synthesizing findings across species, and results from such studies cannot be directly extrapolated to human populations. Observational studies, including those by Schuch et al. [13] and Hanon et al. [15], offer real-world perspectives but lack randomization and are susceptible to confounding, which weakens causal inference. In particular, these studies may not adequately control for key confounders such as baseline cognitive function, education level, comorbid conditions, medication adherence, or socioeconomic status. Even when statistical adjustments are made, residual confounding may persist due to unmeasured or inaccurately measured variables. Furthermore, reliance on self-reported medication use in some cases introduces potential recall bias, especially in cognitively impaired individuals. These limitations complicate the ability to isolate the true effect of RAAS-modulating agents on cognitive outcomes. Moreover, the AVEC trial by Hajjar et al. [22] was excluded due to being published only as a protocol without reported outcomes. Finally, publication bias cannot be excluded, as studies with null or negative results may be underrepresented. These limitations collectively underscore the importance of cautious interpretation of the current evidence and highlight the need for future well-powered, methodologically rigorous trials with standardized cognitive endpoints to better define the role of RAAS-targeting interventions in cognitive health.

When compared with existing literature and clinical practice guidelines, the findings of our review offer a complementary perspective. While most major guidelines, such as those from the American Heart Association (AHA) and National Institute for Health and Care Excellence (NICE), emphasize hypertension management as a preventive strategy for cognitive decline [23,24], they stop short of endorsing specific RAAS-targeting therapies for dementia prevention. Our findings suggest that ARBs may confer additional benefits beyond blood pressure control, particularly in populations at risk for vascular cognitive impairment, though these effects require further substantiation in dedicated cognitive trials. The trend in research toward understanding dementia as a multifactorial condition with strong vascular underpinnings aligns with the mechanistic rationale of RAAS modulation [25].

Significant gaps in knowledge remain, which warrant targeted investigation. There is a pressing need for large, well-powered randomized controlled trials that prioritize cognitive endpoints, ideally over longer follow-up periods exceeding 24 months. Future research should stratify participants by genetic markers such as the ACE I/D polymorphism and APOE genotype to clarify the personalized utility of RAAS-targeted therapies. Furthermore, studies focusing on individuals with prodromal dementia or mild cognitive impairment (MCI) may yield more actionable data, as early-stage interventions are likely to be more effective than those initiated in advanced neurodegeneration. Preclinical research should continue exploring the role of AT2 receptor agonists and non-blood-pressure-mediated effects of RAAS inhibition in neuroprotection.

## Conclusions

This systematic review synthesizes current clinical and preclinical evidence on the influence of RASS in cognitive decline and neuropsychiatric outcomes. While the data suggest that ARBs, and to a lesser extent ACE inhibitors, may offer cognitive benefits in specific at-risk populations, the evidence remains heterogeneous and occasionally inconclusive. Preclinical models support a clear pathophysiologic role of RAAS in neuroinflammation and vascular dysfunction, but clinical translation is still limited by variability in study designs and endpoints. Ultimately, RAAS modulation holds promise as a component of future dementia prevention strategies, particularly in vascular and metabolic contexts, but should be validated by well-structured, cognitively focused trials with precision-medicine considerations.
